# A systematic review of serious games in medical education: quality of evidence and pedagogical strategy

**DOI:** 10.1080/10872981.2018.1438718

**Published:** 2018-02-19

**Authors:** Iouri Gorbanev, Sandra Agudelo-Londoño, Rafael A. González, Ariel Cortes, Alexandra Pomares, Vivian Delgadillo, Francisco J. Yepes, Óscar Muñoz

**Affiliations:** ^a^ Economics and Management School, Pontificia Universidad Javeriana, Bogotá, Colombia; ^b^ Public Health Institute, Pontificia Universidad Javeriana, Bogotá, Colombia; ^c^ Engineering School, Pontificia Universidad Javeriana, Bogotá, Colombia; ^d^ Economics and Management Sciences School, Pontificia Universidad Javeriana, Bogotá, Colombia; ^e^ Medicine School, Pontificia Universidad Javeriana, Bogotá, Colombia

**Keywords:** Video games, medical education, evidence-based practice, comparative effectiveness research, review

## Abstract

**Introduction**: The literature shows an optimistic landscape for the effectiveness of games in medical education. Nevertheless, games are not considered mainstream material in medical teaching. Two research questions that arise are the following: What pedagogical strategies do developers use when creating games for medical education? And what is the quality of the evidence on the effectiveness of games?

**Methods**: A systematic review was made by a multi-disciplinary team of researchers following the Cochrane Collaboration Guidelines. We included peer-reviewed journal articles which described or assessed the use of serious games or gamified apps in medical education. We used the Medical Education Research Study Quality Instrument (MERSQI) to assess the quality of evidence in the use of games. We also evaluated the pedagogical perspectives of such articles.

**Results**: Even though game developers claim that games are useful pedagogical tools, the evidence on their effectiveness is moderate, as assessed by the MERSQI score. Behaviourism and cognitivism continue to be the predominant pedagogical strategies, and games are complementary devices that do not replace traditional medical teaching tools. Medical educators prefer simulations and quizzes focused on knowledge retention and skill development through repetition and do not demand the use of sophisticated games in their classrooms. Moreover, public access to medical games is limited.

**Discussion**: Our aim was to put the pedagogical strategy into dialogue with the evidence on the effectiveness of the use of medical games. This makes sense since the practical use of games depends on the quality of the evidence about their effectiveness. Moreover, recognition of said pedagogical strategy would allow game developers to design more robust games which would greatly contribute to the learning process.

## Introduction

The repertoire of computer strategies for medical education is becoming wider with the introduction of e-learning applications, game-based learning, gamification, and mobile learning [1]. A variety of serious games are ever more frequently used in medical education taking into account that medical students are younger and keen on technologies [2]. Increasing interest toward games is evidenced by a growing number of case reports and systematic reviews about the use of games in education [3–7].

Following Bergeron [8], we understand serious games (in what follows, games) as ‘an interactive computer application, with or without a significant hardware component, that has a challenging goal, is fun to play with, incorporates some concept of scoring, and imparts in the user a skill, knowledge or attitude which can be applied in the real world’. Games are called *serious* when they have a pedagogical purpose. We adopt this wide definition of games because our purpose is that of description, and we want it to be as inclusive and useful for practical teachers as possible.

Bedwell [9] postulated nine characteristics that a serious game must have: an action language (a game offers some method of communication between the person and the game); assessment (tracks the number of correct answers); conflict or challenge; control, or the ability for the players to alter the game; environment; game fiction or story; human interaction among the players; immersion in the game; and rules and goals of the game provided to the player. This definition covers a wide range of products. On the one hand, there is a wide range of video games, and on the other hand, there are gamified e-books or virtual patients with at least one of Bedwell’s attributes.

Games are attractive because they do something that traditional teaching methods do not. The conventional lecture-based teaching emphasizes on information transmission and memory. Games are different since they confront students with an engaging problem and offer possible ways to explore the problematic situation. This way, students have the opportunity to develop higher levels of learning, such as application and analysis [10]. Games have a feedback mechanism and can be designed with a range of levels of difficulty. Trial and error in games has no fatal consequences, and it serves to build up professional skills and team work [3,11]. In the problem-solving process, learning happens when students themselves build their own concepts.

If the promise of learning through games is so attractive, games could change the essence of medical pedagogy. While reflecting on this promise, we found that the literature had not put two key factors into dialogue: the pedagogical perspectives that support the use of games, if any, and the quality of the evidence on the games used.

The educational effect of games may be explained from different pedagogical perspectives: behaviourist, cognitive, humanist, and constructivist. According to behaviourists, learning occurs through operant conditioning; behaviourism prioritizes knowledge transmission. According to cognitivism, learners not only absorb information; instead, they are information processors, and their minds are ‘black boxes’ that need to be understood. Humanism proposes a person-centred learning based on values and intentions and advocates experiential learning. Finally, constructivism highlights knowledge construction through problem-solving and interaction in the social world [12].

Therefore, a selection of a pedagogical perspective would not be indifferent in this case since it mirrors the intentions of game authors and determines the architecture of the games. However, the connection between pedagogical perspectives and serious games is weak. Game developers are more concerned with the practical aspects of their games and neglect their theoretical foundations. Based on what is published in the literature, practicality seems to be emphasized in game development more than theoretical foundations. Thus, a research question arises: When creating games for medical education, what pedagogical strategies did the developers use?

Another concern about using games is the uncertain evidence of the effects of games on learning. Despite the fact that papers on game implementation show an optimistic landscape for the effectiveness of games, systematic reviews are more cautious. Graafland [3] looked for games published between 1995 and April 2012 and found that game developers paid little attention to game effectiveness validation. He used some proprietary qualitative criteria to assess the quality of the evidence reported by game authors. Akl [4] reviewed the articles published before 2007. He found no evidence to neither confirm nor refute the usefulness of educational games as an effective teaching strategy for medical students. He used the qualitative scale EPOC [13]. This scale is better suited to assess the quality of public health interventions than the methodological quality of published articles. Abdulmajed [6] reviewed five games published in 2002–2010 and did not come to any definitive conclusion related to the effectiveness of games. He did not specify the method used to assess the quality of evidence.

The lack of clarity as to the effectiveness of the use of games is echoed by those teachers who have no intention to abandon their traditional lectures or to design their curriculums based on games [4]. A second research question arises: What is the quality of the evidence on the effectiveness of the games?

In order to answer these two questions, we conducted a systematic review of articles on games implemented in medical education. This paper tries to guide teachers in their selection of games based on the best evidence available, and it attempts to make developers aware of the importance of the pedagogical strategy during game design.

## Methods

We conducted a systematic review in accordance with the Cochrane Collaboration guidelines. Since games for medical education stay at the intersection between medicine, pedagogy, and technology, a multi-disciplinary team was needed.

## Inclusion criteria

**Figure 1.**
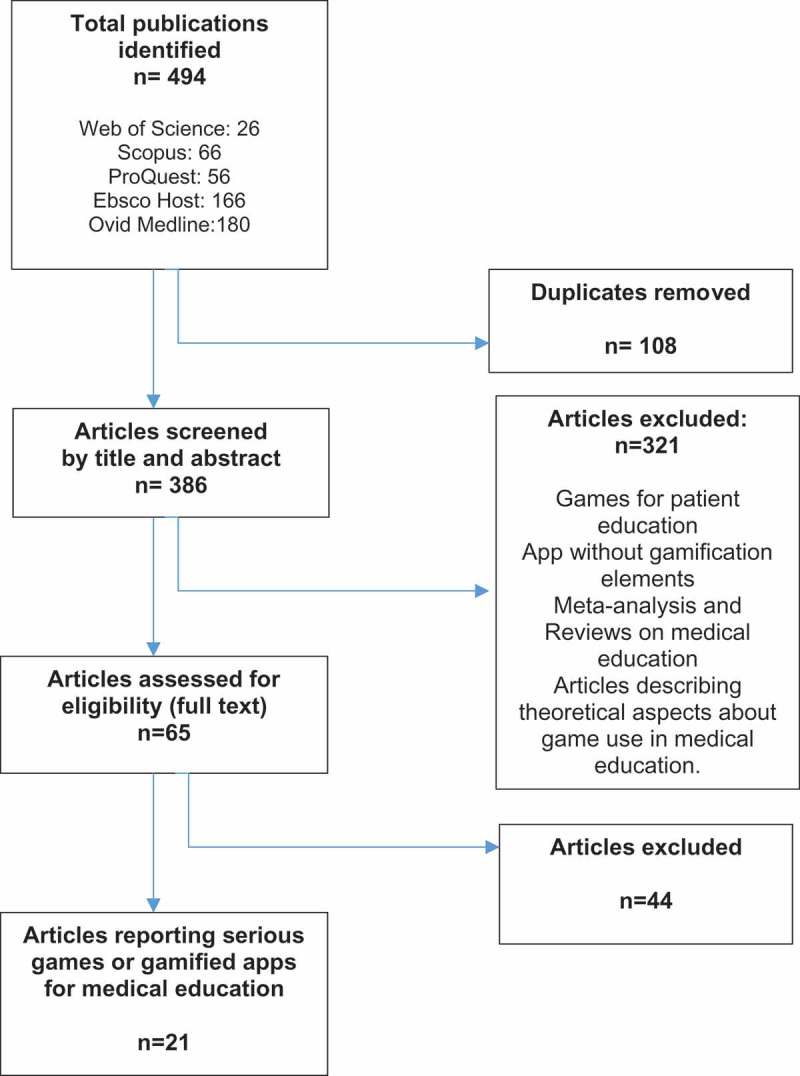
Selection process of articles.

We included peer-reviewed journal articles which described or assessed the use of serious games or gamified apps in medical education. We defined medical education as that exclusively oriented to under and post-graduate-level medical students and doctors, excluding nurses, physical therapists, pharmacologists, and others involved in patient care. We excluded games for patient education, apps without gamification elements, meta-analysis and systematic reviews on games in medical education, and articles describing theoretical aspects about game use in education as indicated in Figure 1.

The time frame for the analysis in English, Spanish, and Portuguese was between 2011 and 2015. This 5-year frame was established due to the high speed of innovation and obsolescence in games.

## Search strategy

Web of science, Scopus, ProQuest, Ebsco Host, and OvidMedline were queried. We built search criteria using DeCs and MeSh terms. These terms were refined using keywords of published articles and an iterative process guided by consultation with a research librarian from the University. Search terms were connected using the Boolean Operators ‘AND’ and ‘OR’ to capture all relevant article suggestions.

Search terms used are computer-based, medical education, technology-enhanced, medical students, learning, physicians, e-learning, education, m-learning, mobile phone, smartphone, mobile app, app$, game*, serious games, gamification.

## Selection process

We screened the databases for reports on game use in medical education based on the title. Those duplicated were excluded. The eight researchers were split into pairs; both members read the abstracts of assigned articles separately and then discussed their findings. If they agreed, the article followed the process. If not, the article was presented to all researchers for further discussion and decision. Then, we read the rest of full papers.

## Data collection

We built a data extraction form in Microsoft Excel®. This form was divided into three categories: (1) study identification, (2) analysis of teaching/learning strategies related to game characteristics, and (3) study design.

The first section contained bibliographic references of the articles, country where the game was created, and demographic description of participants. The second section described aspects related to teaching strategies, students and teacher roles, relation between technology, pedagogical strategy, and learning objectives [14–17].

The study design section evaluated the scientific method of the articles. We found three scales to evaluate medical education research: (1) Medical Education Research Study Quality Instrument (MERSQI), (2) Best Medical Education Evaluation global scale, and (3) Newcastle–Ottawa Scale. These instruments are partly based on Kirkpatrick’s hierarchy of educational outcomes, which provides a valuable conceptual framework for planning and evaluating educational initiatives.

We chose MERSQI [18,19] because it allows assessing the methodological rigor of articles, it includes a comprehensive list of review items, and it also has a growing body of validity evidence [20]. MERSQI adopts Kirkpatrick four-level model to approach the effectiveness construct. The first level (reaction) focuses on the participants’ perceptions of the intervention. The second level (learning) evaluates knowledge, skills, and attitudinal change. The third level (behaviour) measures changes in behaviour. The fourth level (results) focuses on the organization benefits as a result of the intervention [21].

We used Landers [22], Starks [23], Conolly [24], and De Lope [25] in order to build game attributes: type, platform, and game genre.

We used Wu [12] for classifying papers according to the pedagogical strategy. Each paper was read by a pair of reviewers. When they disagreed on the type of pedagogy used in the game, they appealed to a third reviewer in order to decide on this matter. In order to establish game/pedagogy coherence, we took the definition of every strategy by Wu [12] and related it to game attributes.

The data extraction form was piloted with three articles at random. We found that some variables (i.e., pedagogical strategy) were not explicitly stated in the articles. Thus, we decided not to collect these data in a standardized way but to allow the researchers to use a free text format. After adjusting the form, it was applied to all 21 selected studies. We conducted a double review of the abstracts and full-text articles.

## Data analysis

We used descriptive statistics Microsoft Excel® to process data from two viewpoints: game and MERSQI domain perspectives. First, we calculated MERSQI score for each article. Second, we calculated average scores between all the games for each MERSQI domain. Additionally, some qualitative interpretations of pedagogical aspects were made.

## Ethical approval

The project was approved by the Authors’ University Ethics Committee.

## Results

We identified 494 articles from a primary search in the databases. One hundred and eight duplicated articles were excluded. The remaining 386 articles were distributed among pairs. Three hundred twenty-one articles did not meet the inclusion criteria and were excluded. Of the remaining 65 articles, 44 met exclusion criteria and 21 were left for final revision (Figure 1).

All the authors of articles reported some positives effects of games on learning (knowledge and skills) and on students’ motivation. The degree of these effects and the level of scientific evidence varied. The MERSQI scale allowed us to identify that the quality of the evidence provided by the articles was moderate (Table 1).

**Table 1.** MERSQI domain and item scores for 21 studies of games used for medical education.

Domain	Item (score)	MERSQI* Average score (SD)	N° Studies (%)
Study design	**1. Study design**		
	Randomized controlled trial (3)	12.6 (1.2)	9 (42.8)
	Nonrandomized 2 group (2)	11 (0.7)	2 (9.52)
	Single group pre-test and post-test (1.5)	9 (4.9)	2 (9.52)
	Single group cross-sectional or single group post-test only (1)	9 (2.6)	8 (38)
Sampling	**2. No. of institutions studied**		
	>2 institutions(1.5)	8 (0)	1 (4.76)
	2 institutions (1)	10.5 (0)	1 (4.76)
	1 institution (0.5)	10.9 (2.8)	19 (90.4)
	**3. Response rate %**		
	>75 % (1.5)	11.5 (2.2)	17 (80.9)
	50-74 % (1)	8 (3.1)	3 (14.2)
	<50% or not reported (0.5)	6 (0)	1 (4.76)
Type of data	**4. Type of data**		
	Assessment by study participant (1)	7 (2.5)	5 (23.8)
	Objective measurement (3)	11.9 (1.4)	16 (76.1)
Validity of evaluation instrument	**5. Internal structure**		
	Not reported (0)	10 (2.8)	15 (71.4)
	Reported (1)	12.5 (1.5)	6 (28.5)
	**6. Content**		
	Not reported (0)	10 (2.6)	16 (76.1)
	Reported (1)	13 (1.6)	5 (23.8)
	**7. Relationships to other variables**		
	Not reported (0)	10.3 (2.7)	18 (85.7)
	Reported (1)	13 (2.2)	3 (14.2)
Data analysis	**8. Appropriateness of analysis**		
	Data analysis appropriate for study design or type of data (1)	11.3 (2.0)	19 (90.4)
	Not statistical analysis (0)	5 (0.7)	2 (9.52)
	**9. Complexity of analysis**		
	Beyond descriptive analysis (2)	12.1 (1.2)	15 (71.4)
	Descriptive analysis only (1)	8.37 (1.8)	4 (19)
	Not statistical analysis (0)	5 (0.7)	2 (9.52)
Outcomes	**10. Outcomes**		
	Satisfaction, attitudes, perceptions, opinions, general facts (1)	7 (2.5)	5 (23.8)
	Knowledge, skills (1.5)	11.9 (1.4)	16 (76.1)
	Behaviours (2)	0 (0)	0 (0)
	Patient/health care outcome (3)	0 (0)	0 (0)

MERSQI* by Reed (2007) medical education research study quality instrument. SD: Standard deviation

Nine articles used a randomized controlled trial (RCT) (42.8%) to test effectiveness. The second most frequent study design was a single-group cross-sectional or single-group post-test only with eight articles (38%). RCTs obtained the highest MERSQI mean score (12.6 points), and the single cross-sectional or post-test group obtained the lowest score (9 points).

When we assessed outcomes following Kirkpatrick’s criteria used by MERSQI, we found 16 studies focused on knowledge and skills (76.1%); 5 studies focused on satisfaction, attitudes, perceptions, opinions, or general facts (23.8%). No study reported behavioural change or patient/health-care outcomes as a result of game implementation.

We observed that most game projects (90.4%) were implemented at only one institution. In order to evaluate the effectiveness, quantitative tools, like surveys, and qualitative tools, like open-ended questions and focus groups, were used. Only 28.5% of the instruments had internal validity tests.

Despite the methodological limitations of studies, the authors used appropriate statistical analysis (90.4%) according to MERSQI [18]. Also, 76.1% of the studies reported an objective measurement of data. Finally, 80.9% of the studies had good response rates higher than 75%.

According to Table 2, the maximum MERSQI score allowed is 18 points. The articles averaged 10.8 points; the highest score for an article was 15.5, and the lowest was 4.5.

**Table 2.** Evidence on the effectiveness of games.

Author	Author´s Institution	Study design	Specialty	Assessment	N° of participants	Setting	MERSQI score
Boeder (2013) (28)	U. of Munich, Germany	Single group cross-sectional or single group post-test only	Internal medicine	Knowledge	11	Classroom	4.5
Boeker et al (2013)(29)	Freiburg U., Germany	Randomized controlled trial	Urology	Knowledge and perception	145	Classroom	15.5
Yu et al (2015) (30)	U. of Toronto and St. Michael’s Hospital, Canada	Single group cross-sectional or single group post-test only	Physiology/anatomy	Knowledge	75	Hospital or Clinical setting	11
Cendan et al (2011) (31)	U. of Central Florida, United States	Randomized controlled trial	Physiology/anatomy	Knowledge and perception	40	Hospital or Clinical setting	13.5
Creutzfeldt (2012) (32)	Karolinska Institutet, Sweden	Nonrandomized 2 group	Emergency	Skill	30	Hospital or Clinical setting	10.5
Creutzfeldt et al (2010) (33)	Karolinska Institutet, Sweden	Single group pre-test and post-test	Emergency	Knowledge and perception	12	Classroom	12.5
Gasco et al (2014) (34)	U. of Texas, United States	Randomized controlled trial	Neurology	Skill	26	Not reported	12.5
Kanthan et al (2011) (35)	U. of Saskatchewan, Canada	Nonrandomized 2 group	Pathology	Knowledge	185	Classroom	11.5
Knight et al (2010) (36)	U. of Birmingham and others, United Kingdom	Randomized controlled trial	Emergency	Knowledge, perception and skill	91	Hospital or Clinical setting	12.5
Kreiter et al (2011) (37)	U. of Kansas and U. of Iowa, United States	Single group cross-sectional or single group post-test only	Clinical Lab	Knowledge and perception	Started with 13, ended with 143	Classroom	12.5
Lameris et al (2015) (38)	Radboud U., Netherlands	Single group cross-sectional or single group post-test only	Physiology/anatomy	Knowledge and perception	439	Classroom	9
Longmuir (2014) (39)	U. of California, United States	Single group cross-sectional or single group post-test only	Physiology/anatomy	Knowledge	56	Classroom	6
McGrath et al (2015) (40)	Ohio State U., United States	Randomized controlled trial	Emergency	Knowledge	35	Classroom	12.5
Moreno-Ger et al (2010) (41)	Complutense U. of Madrid, Spain	Randomized controlled trial	Physiology/anatomy	Knowledge	143	Hospital or Clinical setting	11
Nevin et al (2014) (42)	U. of Alabama (UAB) -(UAH), United States	Single group cross-sectional or single group post-test only	Internal medicine	Knowledge and perception	128 at UAB and 24 at UAH	Hospital or Clinical setting	10.5
Rondon et al (2013) (43)	U. of Sao Paulo, Brazil	Randomized controlled trial	Phono audiology	Knowledge	29	Classroom	11.5
Sánchez-Rola et al (2014) (44)	U. of Deusto, Spain	Single group cross-sectional or single group post-test only	Neurology	Knowledge	50	Classroom	8
Schmeling et al (2011) (45)	U. of Müenster, Germany	Single group pre-test and post-test	Forensic	Knowledge and perception. But only perception was reported	29	Hospital or Clinical setting	5.5
Silvennoinen et al (2015) (46)	U. of Jyvaskyla, Finland	Single group cross-sectional or single group post-test only	Surgery	Skill	13	Hospital or Clinical setting	10.5
Stirling et al (2014) (47)	Bond U., Australia	Randomized controlled trial	Physiology/anatomy	Knowledge and perception	85	Not reported	12.5
Holloway et al (2015) (48)	McGill U. and Mount Sinai Medical Center, United States and Canada	Randomized controlled trial	Neurosurgery	Skill	83	Hospital or Clinical setting	12.5

Most games (six) were designed in the USA alone; another game was made in the USA together with a Canadian institution. Three games were designed in Germany, two in Sweden, two in Canada, and two in Spain. Despite the fact that we tried to find games published in Spanish or in Portuguese, we could not find any. Only one game was produced in Latin America.

Among the 21 articles, 61.9% did not report any average age of participants. Four articles made tests with students between 20 and 30 years old; four articles reported an age of participants over 30 years old (data not shown). According to the type of participants, 61.9% of them were undergraduate medical students and 28.6% were residents; and one study reported students and physicians as participants.

Games were developed in the following medical specialties: phono audiology, laboratory, surgery, forensics, pathology, neurosurgery, and urology, with one game each. There were four games in the emergency room and two games in the neurology and internal medicine areas. Six games were developed for physiology and anatomy.

Most games were designed to be used in class settings (65%). As to the outcome assessment, eight articles reported knowledge, eight articles reported knowledge and perceptions, and four focused on skills. One article reported all the three aspects.

The pedagogical basis for the games was not always explicitly stated. Nevertheless, they revealed their pedagogical preferences when disclosing the state of medical education, student’s motivation, and the aims of the game. We determined that 47.6% belonged to a behaviourist field and 28.6% to a cognitivist field; they focus on knowledge transmission and try to make it motivating. The rest of the articles propose some form of student-centred learning focused on problem-solving: 4.8% follow a humanist line and 14.3% a constructivist line. One article did not allow determining the pedagogical strategy.

Game designs were coherent with the pedagogical strategy 76.2% of the times. As for the developerʼs choice of game genre, 61.9% of the games were simulations and 33.3% were quizzes; only one article recreated an adventure scenario.

A percentage of 52.4 of the games used a web-based environment, 14.3% were based on mobile apps, and 33.3% on computers. More than 71.4% of them were designed to play a complementary role in a traditional class as reinforcement and review of topics studied, or as training for exams. The rest of the games were implemented as independent devices, i.e., the game was the centre of the educational process, and there was no need for a lecture (Table 3).

**Table 3.** Pedagogical description of games.

Author	Game name	Type	Platform	Game genre	Games’ pedagogical role	Pedagogical strategy	Game and pedagogy coherence
Boeder (2013) (28)	Mediman	Game	Web-based	Quiz	Independent	Behaviourism	Coherent
Boeker et al (2013) (29)	Uro Island	Game like simulation	Computer-based	Adventure	Independent	Cognitivism	Coherent
Yu et al (2015) (30)	DKA simulator	Game like simulation	Computer-based	Training simulation	Complementary	Behaviourism	Coherent
Cendan et al (2011) (31)	Physiosim	Game like simulation	Web-based	Training simulation	Independent	Insufficient pedagogical description	Insufficient game description
Creutzfeldt (2012) (32)	SimMan	Game like simulation	Web-based	Training simulation	Complementary	Humanism	Insufficient game description
Creutzfeldt et al (2010) (33)	MMVW-CPR	Game like simulation	Computer-based	Training simulation	Complementary	Cognitivism	Coherent
Gasco et al (2014) (34)	ImmersiveTouch simulator	Game like simulation	Computer-based	Training simulation	Complementary	Behaviourism	Coherent
Kanthan et al (2011) (35)	Path to Success	Gamified App	Web-based	Quiz	Complementary	Constructivism	Incoherent
Knight et al (2010) (36)	Triage Trainer	Game like simulation	Web-based	Training simulation	Complementary	Behaviourism	Coherent
Kreiter et al (2011) (37)	LabCAPS	Game like simulation	Computer-based	Training simulation	Complementary	Behaviourism	Coherent
Lameris et al (2015) (38)	Physiomics to the next level	Gamified App	Mobile app	Quiz	Complementary	Cognitivism	Coherent
Longmuir (2014) (39)	Interactive module based in AppCobra	Gamified App	Mobile app	Quiz	Complementary	Constructivism	Incoherent
McGrath et al (2015) (40)	Virtual examination	Game like simulation	Web-based	Training simulation	Complementary	Behaviourism	Coherent
Moreno-Ger et al (2010) (41)	HCT Game-like simulation	Gamified App	Web-based	Training simulation	Complementary	Behaviourism	Coherent
Nevin et al (2014) (42)	Kaizen- IM (Kaizen-Internal Medicine)	Gamified App	Web-based	Quiz	Complementary	Behaviourism	Coherent
Rondon et al (2013) (43)	Anatese 2.0	Gamified App	Computer-based	Quiz	Independent	Constructivism	Insufficient game description
Sánchez-Rola et al (2014) (44)	Mobile NBM	Gamified App	Mobile app	Quiz	Independent	Behaviourism	Coherent
Schmeling et al (2011) (45)	Inmedea Simulator	Game like simulation	Web-based	Training simulation	Complementary	Cognitivism	Coherent
Silvennoinen et al (2015) (46)	Lap MentorTM VR simulator	Game like simulation	Computer-based	Training simulation	Complementary	Cognitivism	Coherent
Stirling et al (2014) (47)	Multimedia ebook for anatomy	Game like simulation	Web-based	Training simulation	Independent	Cognitivism	Coherent
Holloway et al (2015) (48)	NeuroTouch simulator	Game like simulation	Web-based	Training simulation	Complementary	Behaviourism	Coherent

## Discussion

We formulated two questions about the pedagogical strategy and the quality of the evidence on effectiveness of the games. This research allowed us to answer both.

As for the pedagogical strategy, 76.2% of the game authors were classified by the researchers as behaviourists or cognitivists. This was quite logical since they preferred quizzes and simulations and focused on memory and skill development through repetition. This finding makes us think that medical educators focus on this two domains and do not demand for more sophisticated games [14]. We agree with Gros [26] that technologies per se – in this case, games – did not change the teacher’s view and the student’s passive role.

It was not possible to assess the size of the pedagogical effect of games on learning because games developers measured different outcomes, used non-standardized instruments, and focused on diverse areas of medical knowledge. Contrary to Kirkpatrick recommendations, the effectiveness tests assessed neither change in behaviour of participants nor health outcomes of patients [21]. Games developers appreciated positively their games as pedagogical tools and recommended to use games in medical education. However, as Kapp had remarked, if one specific game is good for education, this does not mean that all the games are good for such purpose [27].

We did not detect any standard for providing access to games. Some developers publish the URL to their game and try to sell it; few developers upload games for free public use, and most of them do not explain how to get access to their games. No games were promoted by prestigious publishing houses. Without their help, developers do not have the necessary marketing power to position their games on the market and implement them in more than one institution.

Based on what is published, the weakness of diffusion mechanism explains the lack of repeated implementations of games in different settings. Games were implemented, tested, and reported only once and in only one setting. There were no reports on comparative testing of two similar games in the same place. When game developers made implementation in only one setting, this reduced the quality of evidence in MERSQI scale. According to our findings, games and studies about them are part of a particular teacher’s initiative. Game developers should adopt peer networking as a tool for improving their medical games.

This systematic review allowed us to answer the second research question. We found that while most articles reported some positive influence of games on learners, the quality of the evidence provided by the articles was moderate according to MERSQI. This result confirms Akl [4] and All’s [5] findings.

A study limitation encountered was the variety of games terminology available. We began using MeSh terms exclusively but had to abandon this plan because the literature on games develops quickly and uses proprietary non-Mesh terms. The lack of consensus as to the terminology in games is a serious drawback for the researcher. Any effort to reduce the terminological variety in games is welcome.

Another study limitation was related to the MERSQI outcomes domain. The scale is good for assessing evidence on effectiveness but it makes no differentiation between knowledge and skills, whereas modern pedagogic theories had looked at this differentiation in greater depth [21]. Future work in this domain may develop this feature of the MERSQI scale. In addition, the MERSQI scale does not take into account the statistical power of the studies included, which is necessary in order to establish the levels of evidence well.

Future research is needed to understand the relationship between pedagogical perspective and game designs.

## Conclusions

Behaviourism and cognitivism are the predominant pedagogical strategies among game developers. Games are used as complementary devices for classes. While the authors claim that games are useful pedagogical tools, the evidence on their effectiveness is moderate as assessed by the MERSQI scale.

This paper puts the pedagogical strategy into dialogue with the quality of the evidence on the effectiveness of medical games. This makes sense because the practical use of games depends on the quality of the evidence about their effectiveness. On the other hand, recognition of the pedagogical strategy used would allow for the design of more robust games, which would definitely contribute to the learning process.
